# Neuronal damage using Fluoro-Jade B histofluorescence and gliosis in the striatum after various durations of transient cerebral ischemia in gerbils

**DOI:** 10.1186/cc10910

**Published:** 2012-03-20

**Authors:** JH Cho, CW Park, HY Lee, MH Won

**Affiliations:** 1 Kangwon National University, Chuncheonsi, South Korea

## Introduction

Ischemic damage occurs well in vulnerable regions of the brain, including the hippocampus and striatum. In the present study, we examined neuronal damage/death and glial changes in the striatum 4 days after 5, 10, 15 and 20 minutes of transient cerebral ischemia using the gerbil. Spontaneous motor activity was shown to be increased with the duration time of ischemia-reperfusion (I-R).

## Methods

To examine neuronal damage, we used Fluoro-Jade B (F-JB, a marker for neuronal degeneration) histofluorescence staining. F-JB-positive cells were detected only in the 20-minute ischemia group, not in the other groups. In addition, we examined gliosis of astrocytes and microglia using antiglial fibrillary acidic protein (GFAP) and anti-ionized calcium-binding adapter molecule 1 (Iba-1), respectively.

## Results

In the 5-minute ischemia group, GFAP-immunoreactive astrocytes were distinctively increased in number, and the immunoreactivity was stronger than that in the sham group. In the 10-minute, 15-minute and 20-minute ischemia groups, GFAP immunoreactivity was more increased with the duration of I-R. On the other hand, the immunoreactivity and number of Iba-1-immunoreactive microglia were distinctively increased in the 5-minute and 10-minute ischemia groups. In the 15-minute ischemia group, microglia were largest in size, and the immunoreactivity was highest; however, in the 20-minute ischemia group, the immunoreactivity was low compared to the 15-minute ischemia group. The results of western blotting for GFAP and Iba-1 were similar to the immunohistochemical data.

## Conclusion

These findings indicate that neuronal death was detected only in the 20-minute ischemia group 4 days after I-R; in addition, the change pattern of astrocytes and microglia were apparently different according to the duration time of I-R.

